# Antitumor Immunity Produced by the Liver Kupffer Cells, NK Cells, NKT Cells, and CD8^+^ CD122^+^ T Cells

**DOI:** 10.1155/2011/868345

**Published:** 2011-11-29

**Authors:** Shuhji Seki, Hiroyuki Nakashima, Masahiro Nakashima, Manabu Kinoshita

**Affiliations:** Department of Immunology and Microbiology, National Defense Medical College, Namiki-3-2, Tokorozawa, Saitama 358-8513, Japan

## Abstract

Mouse and human livers contain innate immune leukocytes, NK cells, NKT cells, and macrophage-lineage Kupffer cells. Various bacterial components, including Toll-like receptor (TLR) ligands and an NKT cell ligand (**α**-galactocylceramide), activate liver Kupffer cells, which produce IL-1, IL-6, IL-12, and TNF. IL-12 activates hepatic NK cells and NKT cells to produce IFN-**γ**, which further activates hepatic T cells, in turn activating phagocytosis and cytokine production by Kupffer cells in a positive feedback loop. These immunological events are essentially evoked to protect the host from bacterial and viral infections; however, these events also contribute to antitumor and antimetastatic immunity in the liver by activated liver NK cells and NKT cells. Bystander CD8^+^CD122^+^ T cells, and tumor-specific memory CD8^+^T cells, are also induced in the liver by **α**-galactocylceramide. Furthermore, adoptive transfer experiments have revealed that activated liver lymphocytes may migrate to other organs to inhibit tumor growth, such as the lungs and kidneys. The immunological mechanism underlying the development of hepatocellular carcinoma in cirrhotic livers in hepatitis C patients and liver innate immunity as a double-edged sword (hepatocyte injury/regeneration, septic shock, autoimmune disease, etc.) are also discussed.

## 1. Introduction

The liver is the largest organ in vertebrates. Cumulative evidence has indicated that not only the fetal liver but also the adult liver is an important immune organ. The livers in adult mice contain c-kit^+^ pluripotent hematopoietic stem cells, which are located in the perisinusoidal Disse spaces, and give rise to all lineages of leukocytes and red blood cells [1–3]. c-kit hematopoietic stem cells have also been identified in adult human livers [4]. When B cell- and T cell-deficient SCID mice were lethally irradiated and received bone marrow cells as well as liver mononuclear cells (MNCs) (but not splenocytes) from normal mice, the SCID mice could survive, and the thymus, liver leukocytes, splenocytes, and lymph nodes and bone marrow cells were all reconstituted [1]. The administration of purified c-kit^+^ hematopoietic stem cells from either bone marrow or liver MNCs into SCID mice also reconstituted leukocytes in all organs [1]. In addition, liver Kupffer cells comprise 80% of the macrophage lineage cells in the whole body, and most bacteria that enter the blood stream accumulate in the liver and are killed by these Kupffer cells. In addition, other innate immune lymphocytes, NK cells [5, 6], and T cells with intermediate levels of TCR (TCR^int^ cells) are abundantly present in the liver [7], which are rarely seen in other organs and peripheral blood.

Among mouse TCR^int^ cells in the liver, 2/3 are CD122 (IL-2 receptor *β*)^+^NK1.1^+^ NKT cells and 1/3 are NK1.1^−^CD122^+^ T cells [3, 8–10]. The NK1.1^+^ NKT cells are dependent on an MHC class-I like molecule, CD1d, for their development, express an invariant V*α*14J*α*18/V*β*8 gene product for their T cell receptor (TCR), and have a phenotype of CD4 or CD4^−^CD8^−^(double negative, DN) (afterwards, NKT cells) [10, 11]. On the other hand, NK1.1^−^CD122^+^ T cells are MHC class-I dependent for their development, and predominantly (11%) express the V*α*11 gene product for their TCR [10] and have a phenotype of CD8 or DN (2/3 are CD8^+^ and 1/3 are DN) (afterwards, CD8^+^CD122^+^ T cells). Since CD8^+^CD122^+^ T cells are also present in athymic nude mice and increase age-dependently in nude and normal mice, they may be of extrathymic origin.

Under physiological conditions, most MNCs (including Kupffer cells, NK cells, TCR^int^ cells) exist in the sinusoidal space in the liver parenchyma. Kupffer cells tightly adhere to sinusoidal endothelial cells, and NK/NKT cells are often in contact with these Kupffer cells and may normally elicit immunological functions to eliminate exogenous pathogens present in liver sinusoids that enter from portal vein and the systemic circulation. However, the localization of these MNCs is altered under pathological conditions. In human viral hepatitis or autoimmune hepatitis, a large number of lymphocytes infiltrate into the portal areas (where the portal vein, hepatic artery, and bile duct exist) and cause periportal inflammation. The experimental hepatitis model induced by *α*-galactosylceramide (*α*-GalCer, see Section 3) in mice leads to pathological findings similar to human viral hepatitis, such as piecemeal necrosis and apoptotic Councilman bodies in and around the portal areas, although MNCs also increased in sinusoids. These findings suggest that the antigen activation process may be initiated in and around portal areas.

NKT cells are mainly confined in the liver, and the proportion of NKT cells in liver MNCs remains constant regardless of the age of the mouse, whereas the CD8^+^CD122^+^ T cells constantly increase in the liver, as well as in the periphery, in an age-dependent manner [10]. In addition, both TCR^int^ cells display a potent IFN-*γ* producing capacity and antitumor cytotoxicity [12]. Notably, DN T cells with the intermediate TCR expanded in the liver, spleen, and lymph nodes in autoimmune MRL-*lpr/lpr (lpr) *mice may be an abnormal counterpart to CD8^+^CD122^+^ T cells in the liver of normal mice [7, 10]. Since the Fas (CD95) gene is muted in *lpr* mice [13], it may accelerate the proliferation instead of the apoptosis of activated CD8^+^CD122^+^ T cells in the liver, and they may migrate into periphery after downregulation of CD8 [10].

Bacteria and their components, lipopolysaccharide, peptidoglycan-polysaccharide, and various toxins are physiologically brought from the intestine to the liver [14, 15] and may stimulate these liver leukocytes and their antimicrobial and antitumor immune function. In addition, the IL-6 produced by Kupffer cells/hepatocytes stimulates hepatocytes to produce acute phase proteins (including CRP) and subsequent complement production [16–18]. Therefore, the liver is not only the organ for sugar, protein, and lipid/cholesterol metabolism but also an immune organ. This review focuses on the crucial role of the liver leukocytes in the antitumor and antimetastatic immunity.

## 2. Inhibition of Hematogenous Tumor Metastases in the Liver by NKT Cells Stimulated with Recombinant Interleukin-12 (IL-12)

IL-12 was discovered in both mice and humans around 1990 as an NK cell stimulatory factor [19–21]. IL-12 was initially thought to activate NK cells and cytotoxic CD8^+^T cells to inhibit tumor metastasis. However, we found that the main effector cells that inhibit tumor metastasis of intravenously (i.v.) injected tumors are NKT cells [22–25]. When liver metastatic EL-4 cells (lymphoma), lung metastatic 3LL cells (Louis lung carcinoma), and other tumors were injected into B6 or other strains of mice via a tail vein, the main antimetastatic effectors in the liver, as well as in the lung, were NKT cells (Table 1) [22–25]. However, NK cells were not significantly involved, because IL-12 exerted a potent antimetastatic effect in the liver and lung in NK-deficient beige (bg/bg) mice (Table 1) [23]. In addition, the depletion of both NK cells and NKT cells by anti-NK1.1 Ab, but not the depletion of NK cells alone by an asialo-GM1 Ab, inhibited the IL-12-induced antimetastatic effects in both organs (Table 1) [25]. Furthermore, adoptive transfers of various sorted lymphocyte subsets in liver MNCs from IL-12-injected mice into tumor-inoculated mice confirmed that NKT cells, but not NK cells or CD8^+^T cells, are antimetastatic effectors in the liver, the lungs, and kidneys [3, 24]. These results were further confirmed in NKT cell-deficient mice [26]. However, NK cells and CD8^+^ T cells seem to be effectors against subcutaneous tumor growth [3]. 

Although some researchers have claimed that NKT cells disappear after IL-12 injection by activation-induced apoptosis, and therefore could not be the antimetastatic effectors, we demonstrated that IL-12 merely downregulates NK1.1 expression on NKT cells [27]. NKT cells in IL-12-pretreated mice (24 hours before) were further activated by the injection of a synthetic ligand, *α*-galactosylceramide (*α*-GalCer), and were observed to produce much more IFN-*γ*, as well as IL-4, and to acquire a more potent antitumor cytotoxicity than those in mice without IL-12 pretreatment [27]. It should be noted, however, that IL-12 pretreatment increased TNF receptor and Fas-ligand (FasL) of NKT cells and thereby augmented hepatotoxicity of NKT cells after *α*-GalCer injection [27]. However, as described hereinafter, such hepatotoxicity of *α*-GalCer-activated NKT cells can be completely inhibited by an anti-TNF-Ab without attenuating the antitumor immunity of the NK cells.

## 3. Inhibition of the Tumor Growth in the Liver by *α*-GalCer and Induction of Bystander CD8^+^ CD122^+^ T Cells and Tumor-Specific Cytotoxic CD8^+^ T Cells

*α*-GalCer was initially identified and extracted from a marine sponge, and thereafter synthesized by Kirin Brewery Company [28], and was subsequently observed to strongly inhibit the liver and lung growth of i.v. injected tumor cells. *α*-GalCer was found to be a ligand of the invariant V*α*14J*α*18/V*β*8 TCR of mouse NKT cells [29]. Therefore, NKT cells were initially thought to be antitumor effectors in the liver and lung, but NK cells were also suggested to be antitumor effectors after *α*-GalCer injection. However, the mice injected with *α*-GalCer were shown to have hepatic injury [30, 31]. Thereafter, we demonstrated that the NK cells stimulated with IFN-*γ* produced by *α*-GalCer-activated-NKT cells are the main antitumor effectors, whereas NKT cells themselves are not antitumor effectors, but they do induce hepatotoxicity as a result of their increased FasL expression [31, 32], in which lymphocyte infiltration and apoptotic hepatocytes (Councilman bodies) were observed in and around the portal areas. In addition, although NKT cells were initially thought to disappear due to apoptosis, and thus would not be able to further attack hepatocytes, it was subsequently found that the NKT cells merely transiently downregulated both NK1.1 and TCR [33, 34], in a manner similar to that observed after the injection of IL-12 [27]. These findings suggest that NKT cells downregulate their receptors to inhibit their autoreactivity.

The antitumor function of liver NK cells and the liver injury resulting from NKT cells induced by *α*-GalCer both increase age-dependently [31, 32]. Interestingly, however, when an anti-TNF Ab was injected simultaneously with *α*-GalCer into aged mice after i.v. injection of EL-4 tumor cells or intrasplenic injection of B16 tumor cells, the hepatic injury was completely inhibited, without attenuating the antitumor and antimetastatic activity of the liver NK cells [35].

**Figure 1:**
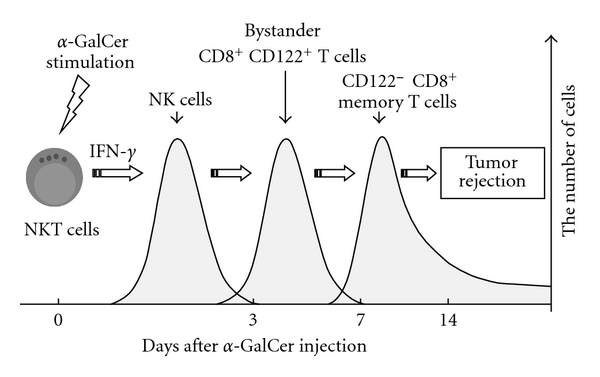
Sequential activation of liver lymphocytes and antitumor immunity by *α*-GlaCer.

The *α*-GalCer-induced NK cells with antitumor activity can kill not only NK-sensitive Yac-1 cells but also NK-resistant B16 cells, EL-4 cells and Colon 26 cells, and can inhibit the liver and lung metastasis of these NK-resistant tumors [36, 37]. Therefore, such activated NK cells may upregulate their killer activating molecules and/or downregulate their killer inhibitory molecules (e.g., CD94/NKG2A) as described in Section 4. Furthermore, after the activation of NK cells, bystander CD8^+^CD122^+^TCR^int^ cells and tumor-specific memory CD8^+^T cells were induced after *α*-GalCer injection, thus allowing the mice to survive. Therefore, if such memory is achieved against certain tumors (e.g., B16 cells), these mice can reject subcutaneously rechallenged B16 cells but cannot reject other tumors (EL-4, Colon-26, etc.) [38]. Following NK cell activation for 2 to 3 days after *α*-GalCer injection, bystander CD8^+^CD122^+^cells with NK cell-like antitumor activity without tumor-specificity are increased at 3 to 7 days after *α*-GalCer injection, while memory CD8^+^T cells, which are cytotoxic only against certain tumors, are induced within two weeks (Figure 1). 

Clinical trials using i.v. transfer of *α*-GalCer-pulsed DCs or PBMCs stimulated with *α*-GalCer in vitro for patients with advanced nonsmall cell lung cancer have been reported. In one report, *α*-GalCer-pulsed PBMCs cultured with IL-2 and GM-CSF were injected into patients four times, and the patients with increased IFN-*γ* producing cells in the PBMCs showed a longer survival (31.9 months, *n* = 17) than the poor responder patients (9.7 months, *n* = 7) [39]. Although no severe adverse event related to the treatment was observed, among several clinical trials, there was no case of obvious tumor regression [39], and a further evaluation of the survival benefit of such immunotherapy is required. It should also be noted that *α*-GalCer-reactive (specific) NKT cells are rare in humans as described in Section 5.

## 4. Antitumor Immunity in the Liver Induced by Bacterial Reagents

### 4.1. Lipopolysachharide (LPS)

When mice were intraperitoneally (i.p.) or i.v. injected with a gram negative bacteria component, LPS, Kupffer cells were activated via toll-like receptor- (TLR-) 4 [40] and produced IL-12, which stimulated NK cells to produce IFN-*γ* and activated NKT cells to acquire potent antitumor cytotoxicity [41]. As noted in Section 1, exogenous IL-12 injection stimulates the IFN-*γ* production and antitumor cytotoxicity of NKT cells, whereas NK cells are not main IFN-*γ* producers nor enhance their antitumor cytotoxicity. However, in the case of LPS injection, NK cells are the essential IFN-*γ* producers, while NKT cells are the main antitumor effectors [3]. This relationship between NK cells and NKT cells after LPS injection is opposite to that after *α*-GalCer injection (Table 2). Therefore, the IFN-*γ*-producing cells and final antitumor effectors differ based upon the stimulating reagent, whereas Kupffer cells are a constant provider of IL-12 [3].

The Kupffer cells activated by LPS also produce IL-6, which stimulates hepatocytes to produce acute phase proteins (including CRP) and complement components [3]. CRP stimulates Kupffer cells via Fc*γ* receptor II and enhances their phagocytic activity [42]. Since a small amount of LPS is considered to be continuously brought to the liver from the intestines via portal vein, such an environment in the liver induces a predominant presence of NK cells and NKT cells in the liver sinusoids [3]. In fact, when mice are maintained under the conventional condition, the number of liver MNCs, including NK cells, NKT cells, and CD8^+^ CD122^+^ T cells, are increased up to 2-fold compared to the numbers in mice maintained under SPF conditions, especially in aged mice [43]. Although LPS injection into mice triggers substantial antitumor immunity in the liver against liver metastatic tumors (EL-4 cells, etc.), in contrast to IL-12, LPS exerts antimetastatic effects only when injected before, but not after, tumor inoculation [41]. It is suggested that LPS, but not IL-12, induces potent TNF production from Kupffer cells/macrophages, which may induce adverse effects on the host defense, especially in tumor-inoculated mice. In fact, TNF reportedly increased tumor metastasis to the lungs [44].

### 4.2. Streptococcal Reagents

It has been well documented that when a *Streptococcus pyogenes* derivative (OK432) is injected to mice, the liver NK cells are increased and activated, and they suppress tumor metastasis in the liver [45, 46] (Table 2). T cells and NKT cells are not likely involved in this antitumor effect, because depletion of NK cells alone by an antiasialo GM1 Ab greatly diminished the antimetastatic effect of OK432. Since *Streptococcus pyogenes *is a gram positive bacteria that lacks LPS, either the teichoic acid, peptidoglycan-polysaccharide, or DNA motifs of *Streptococcus pyogenes* may stimulate Kupffer cells to produce IL-12 either through TLR-2 (teichoic acid, peptidoglycan-polysaccharide) or TLR-9 (bacterial DNA).

### 4.3. Bacteria DNA Motifs (CpG-ODN)

**Figure 2:**
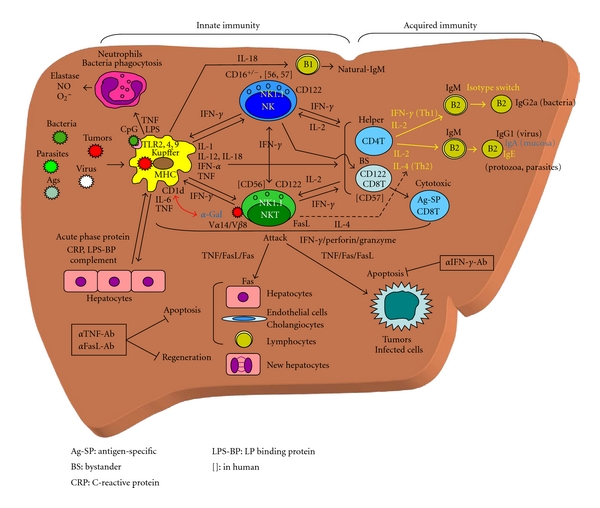
Scheme of immune responses in the liver.

CpG-ODN (oligodeoxynucleotides; GACGTT for mouse, GTCGTT for humans) has been shown to activate innate immunity via the TLR-9 expressed by macrophages [47–49]. This is an important finding, because these DNA motifs are common in all bacteria, and every bacterial infection or invasion can activate innate immunity in both humans and mice [49]. The differences in the frequency of unmethylated CpG dinucleotides between bacterial and vertebrate DNA provide a structural characteristic through which vertebrate immune cells are activated and respond to a bacterial infection [47, 49]. The CpG-ODN thus mimics the stimulatory effect of the DNA of either gram-negative of gram-positive bacteria. When CpG-ODN was injected into mice, the mouse Kupffer cells produced IL-12 and TNF and activated NK cells, as well as NKT cells in the liver (Figure 2).

 Interestingly, IL-12-activated NK cells showed antitumor cytotoxicity after CpG-ODN injection, whereas NKT cells activated by TNF induced hepatocyte injury by expressing FasL [50]. Although the antitumor cytotoxicity and IFN-*γ* production of NK cells is attenuated with aging, the TNF production from Kupffer cells and FasL expression and hepatotoxicity of NKT cells are both augmented with aging [50]. The antitumor activity of CpG-ODN-stimulated NK cells may also be mediated by interferon-*α* [51], and the IFN-*α* production was also decreased with age [50]. Again, although the three bacterial reagents described above all activate Kupffer cells to produce IL-12, it is not clear at present why NKT cells are the main antitumor effectors induced by LPS, while NK cells are the main antitumor effectors induced by Streptococcal derivative and CpG-ODN (Table 2). A further study is needed to address this issue.

It should be noted that although several bacteria and their components have been suggested to be a natural ligand of NKT cells, we feel that certain bacteria or their components are not likely to be a ligand of NKT cells. As described above, activation of NK/NKT cells by LPS or CpG-ODN suggests that every gram positive or negative bacterium can indirectly activate NKT cells. Furthermore, major effectors to fight against bacteria are macrophages and neutrophils.

## 5. Antitumor Cytotoxicity of Human CD56^+^T Cells, CD16^+^CD56^+^NK Cells, and CD16^−^CD56^++^ NK cells

It has been proposed that human NKT cells could be T cells bearing V*α*24J*α*18/V*β*11 gene products for their TCR, because their TCR genes show sequence homology with the mouse TCR V*α*14J*α*18/V*β*8 genes of NKT cells. In addition, both such T cells in mice and humans are specifically activated and proliferated by stimulation with *α*-GalCer. However, we demonstrated that V*α*24J*α*18/V*β*11^+^ T cells are very rare in human peripheral blood and liver MNCs [3, 52]. Even in the liver MNCs, they occupy less than 0.5% of T cells, and we proposed that CD56^+^ T cells (mostly CD8^+^) are the human counterpart of mouse NKT cells. The reasons are as follows. (i) Human liver MNCs contain 25% CD56^+^ NK cells and 20% CD56^+^ T cells, similar to mouse liver NK cells and NKT cells [53]. (ii) The CD56^+^ T cells vigorously proliferate and are activated after stimulation with IL-2 and IL-12 and acquire potent antitumor cytotoxicity [53, 54]. (iii) CD56^+^ T cells have intermediate and pauciclonal TCRs similarly to mouse NKT cells [55]. The NKT cells and NK cells therefore likely play an important role in preventing tumor growth and metastases in the human liver as well as in mouse liver.

Most human NK cells in peripheral blood mononuclear cells (PBMCs) are CD16^+^CD56^+^ NK cells (10~15% of PBMC), while a small number of CD16^−^CD56^++^ NK cells, which express higher levels of CD56 than conventional CD16^+^CD56^+^ NK cells, are present (approximately 1% in PBMCs and 10% of NK cells) [56–58]. Although CD16^−^CD56^++^ NK cells are far less cytotoxic than CD16^+^CD56^+^ NK cells in their resting state, when purified and stimulated with IL-2, IL-12, and IL-15 for several days, the CD16^−^CD56^++^ NK cells proliferate more vigorously compared to CD16^+^CD56^+^ NK cells, and some CD16^−^CD56^++^ NK cells acquire CD16 expression. These CD16^+^ CD56^++^ NK cells produce a large amount of IFN-*γ* and display strong antitumor cytotoxicities against not only NK-sensitive K562 cells but also NK-resistant Raji cells [58–61]. These cells are also induced by Streptococcal derivative and heat-killed *Streptococcus* from PBMC or CD16^−^CD56^++^ NK cells [58]. Although most of these cells express NKG2A (an NK-inhibitory receptor), they also express NKG2D (an NK-activating receptor) and other natural cytotoxicity receptors (NKp30, NKp44, and NKp46) and therefore can kill NK-resistant tumors [58]. Interestingly, the majority of NK cells in the liver, colon, lymph nodes, uterus, and placenta are CD16^−^CD56^+^ NK cells [58, 62]. Therefore, these cells in the human liver, when activated, may have the potential to produce IFN-*γ* and kill various tumors. It can be speculated that NK cells in PBMCs are moving in the rapid blood flow in vessels and can therefore monitor pathogens and tumor cells that invade the blood stream. They need to have the NK activity to immediately attack virus-infected cells and malignant cells and express CD16 (Fc*γ*RIII), presumably for induction of antibody-dependent cell-mediated cytotoxicity (ADCC) of infected cells, microbes, and tumors.

On the other hand, since NK cells in organs do not usually encounter pathogens, they do not need to be in an activated state. However, when once a pathogen/bacteria invaded the organs, they need to be activated to reject the pathogens. However, together with NKT cells, tissue macrophages, and neutrophils, these cells sometimes induce tissue damage and multiorgan dysfunction (MODS) as a result of their autoreactivity, as is the case in septic shock. Therefore, in order to reduce tissue damage, they are thought to normally be in resting states. It should be noted that mouse counterpart of human CD16^−^CD56^++^ NK cells cannot be identified because mouse NK cells do not express CD56. However, since activated NK cells induced by *α*-GalCer, CpG-ODN, or a Streptococcal derivative can kill NK-resistant tumors, similar NK cells may also exist in mice. Whether these CD16^−^CD56^++^ NK cells and CD16^+^ CD56^+^ cells with NK activity are the same lineage cells or distinct subsets needs further investigation.

## 6. CD16^−^CD56^++^ NK Cells in Diseases and in the Clinical Setting

As described perviously, CD16^−^CD56^++^ NK cells and their production of IFN-*γ* may play an important role in antitumor immunity; however, the expansion of CD16^−^CD56^++^ NK cells has been observed in some diseases and in the clinical setting. These cells are the first lymphocytes to appear in the PBMCs after bone marrow transplantation [63]. These cells are also reportedly expanded in the PBMCs of patients with systemic lupus [64], in the synovial fluid of patients with rheumatoid arthritis, and in patients with autoimmune hepatitis [65]. In addition, as described above, these cells were found to expand in vitro after stimulation of PBMCs with a *Streptococcus pyogenes* reagent (OK-432) [58], suggesting their involvement in bacterial infections. In contrast, the number of CD56^++^ NK cells was decreased in the PBMCs of patients with allergic rhinitis and/or asthma [66], suggesting their role in Th1 but not Th2 immune responses.

It has recently been reported that liver CD56^+^ NK cells (presumably CD16^−^ cells) were increased in the livers of primary bilially cirrhosis (PBC) patients. These cells are frequently seen in the portal area, within the biliary epithelium, and around bile ducts [67]. NK cells from the PBC livers stimulated with a combination of TLR-4 and TLR-3 ligands (LPS and Poly I:C, resp.) in vitro exhibited a higher cytotoxic activity against autologous primary human biliary epithelial cells (cholangiocytes) than liver MNCs from subjects with other liver diseases (viral hepatitis and alcoholic liver disease), in which IFN-*α*-produced Kupffer cells stimulated by the TLR-3 ligand may also be required [67]. These findings suggest an important role for CD56^+^ NK cells in PBC. Regarding NK and NKT cells in autoimmune diseases, it should be noted here that NKT cells in mice and humans reportedly inhibit autoimmune diseases (systemic lupus, experimental encephaolomyelitis, Type I diabetes, etc.). However, the role of NKT cells in autoimmune diseases should be carefully evaluated, because NKT cell activation by *α*-GalCer conversely accelerated the onset of lupus-like symptoms, autoantibody production, and hepatotoxicity in NZB/W mice [30, 68]. Further, the effect of *α*-GalCer depends on the mouse strains being examined [69].

Overall, these findings suggest that CD16^−^CD56^++^ NK cells, together with conventional NK cells, NKT cells, and Kupffer cells may play significant roles in Th1 immune responses against cancers and infections, in some autoimmune diseases, and also presumably in nonalcoholic steatohepatitis (NASH).

## 7. The Effects of Aging on Antitumor Immunity, Septic Shock, and MODS

The *α*-GalCer-induced antitumor immunity in the liver (antitumor cytotoxicity) produced by NK cells and the MODS induced by NKT cells unexpectedly both increases with age [32]. In general, antitumor immunity in the liver and other organs appears to decrease with aging, although the proportions of CD57^+^ T cells (a human counterpart of mouse CD8^+^CD122^+^ TCR^int^ cells) and NK cells increase with aging [54]. Consistently, CpG-ODN-induced antitumor immunity and IFN-*γ* production from liver NK cells decreases age-dependently [50]. The septic shock and MODS in mice induced by CpG-ODN administration [70] also worsened age-dependently, because macrophages/Kupffer cells produce a large amount of TNF, and NKT cells increase their FasL expression [50]. The septic shock induced by IL-12 and low-dose LPS (16 h apart) is called the generalized Shwartzman reaction (GSR) and the GSR is also aggravated with aging, because CD8^+^CD122^+^ cells with IFN-*γ* producing capacity and the TNF production by macrophages/Kupffer cells (final effectors for MODS) both increase age-dependently [71]. Thus, liver innate immunity can be a double-edged sword.

Using human PBMCs, an in vitro GSR-like phenomenon can also be reproduced when the PBMCs are stimulated with IL-12 and LPS (24 h apart), because NK cells and CD57^+^ T cells with IFN-*γ* producing capacity increase with age, and the TNF production from macrophages also increases with age [72]. These results explain why septic shock after abdominal surgery occurs more frequently in elderly patients [72]. Thus, innate immunity is a double-edged sword, and aging attenuates the antitumor anti-microbial immunity but aggravates tissue damage. Tissue damage or MODS can be avoided by the administration of an anti-TNF-Ab [35, 50], but the occurrence of any side effects (bacterial infection, especially tuberculosis) should be carefully monitored. In this regard, synthetic CRP may be an effective modulator of innate immunity, which enhances the phagocytic actvity of Kupffer cells and reduces their TNF production, without attenuation of IFN-*γ* production from NK/NKT cells [42]. In fact, the administration of synthetic CRP improved the survival of the mice from bacterial infections and GSR [42].

## 8. The Role of Liver NKT Cells and NKT Cells in Hepatitis C Cirrhosis Patients and the Development of Hepatocellular Carcinoma (HCC)

We previously demonstrated in hepatitis C patients that NKT cells (CD56^+^ T cells), and subsequently, CD56^+^ NK cells, constantly decrease as hepatitis C progresses to cirrhosis, and most of NKT cells and NK cells are lost in cirrhotic livers [52]. Consequently, when liver MNCs obtained from surgical liver specimens of cirrhosis patients with HCC were cultured with IL-2, IL-12, and IL-15, they showed decreased IFN-*γ* production and antitumor cytotoxicity against both K562 cells and Raji cells, which was also the case against an HCC cell line (HuH-7 cells) [52]. Liver NK cells can kill MHC class-I (−) K562 cells, but not MHC class I (+) Raji cells, because MHC class-I molecules inhibit NK cell cytotoxicity by inhibitory signaling, while NKT cells effectively kill Raji cells, but not K562 cells. Interestingly, since HuH-7 cells express low levels of MHC class-I, cytokine-activated NK cells more effectively kill HuH-7 cells than NKT cells [52]. These results suggest that the decrease of NK cells, as well as NKT cells and their antitumor activities, is an important immunological mechanism that may allow the development of HCC in hepatitis C-associated cirrhotic livers. It was also reported in mice that NKT cells were lost in CCL4-induced cirrhotic livers [73]. These results suggest that maintenance of NKT cells in the liver requires normal organization of liver parenchyma. However, notably, if CD94/NKG2A (inhibitory receptors) were blocked by an antibody, NK cells could effectively kill MHC class-I (−) tumors [54]. In addition, since most liver NK cells are CD16 negative and can be activated by cytokines produced by Kupffer cells (IL-12, etc.) and may express NKG2D and other activating molecules, they can kill class-I (−) tumors. These findings suggest that the relationship between NK cells (CD16^+ or −^)/NKT cells and tumor cells in the liver during antitumor immunity is more complex than previously expected.

Although the functional impairment of NK cells and NKT cells may also play an important role in the development of HCC in hepatitis B patients, we could not find any decrease in CD56^+^ T cells and NK cells in the livers of HCC patients with hepatitis B (our unpublished observation), suggesting that the behavior of lymphocytes in hepatitis C and hepatitis B may be different. It is known that, although most HCC cases develops in cirrhotic livers with hepatitis C, HCC also develop in livers with hepatitis B patients without apparent cirrhosis.

## 9. Possible Interactions of TNF, NKT Cells, and FasL with Hepatocytes

As described previously (Sections 3 and 4), although both *α*-GalCer and CpG-ODN induce antitumor activity by hepatic NK cells, they also activate NKT cells to induce hepatocyte injury through the TNF/FasL/Fas pathway [31, 32, 50]. In this regard, it has been unclear whether NKT cells express FasL only to damage hepatocytes, or whether there is a protective function. An important finding was that both *α*-GalCer and CpG-ODN induce hepatocyte injury in aged mice, but not in young mice [32, 50]. Furthermore, *α*-GalCer-activated NKT cells accelerate hepatocyte and liver regeneration after 70% partial hepatectomy (PHx) in mice, which is also TNF/FasL-dependent, whereas NK cells are inhibitory to liver regeneration [74]. In Fas-mutated autoimmune *lpr* mice and NKT cell-deficient CD1d−/− mice, and in normal B6 mice depleted of TNF or FasL by neutralizing Abs, there was no accelerated regeneration of the PHx liver after *α*-GalCer injection [74] (Figure 2). Consistent with these results, it was reported that injection of exogenous TNF or anti-Fas Ab into PHx mice accelerated the regeneration of the PHx liver [75–77]. These findings suggest that NKT cells may normally regulate the turnover of hepatocytes (newly generated hepatocytes and old hepatocytes), the normal lifespan of which is around 200 days [78]. Hepatocytes nascent at the portal space gradually stream toward the terminal hepatic vein, where they are probably eliminated by apoptosis [78]. However, since most HCC shows reduced Fas expression in both hepatitis B and C patients [79–81], HCC may develop by evading surveillance of FasL-expressing NKT cells.

## 10. The Role of NK Cells, NKT Cells, and Kupffer Cells in the Development of Liver Metastasis of Colon Cancers

Malignant tumors, especially those of the colon and stomach, metastasize to the liver via the portal vein. Several experimental studies in mice and rats have demonstrated that NK cells are important antimetastatic effectors in the liver. NK cells are located in the liver sinusoids and adhere to sinusoidal endothelial cells and Kupffer cells, which bind to colon tumor cells injected from mesenteric veins, and kill them. Since anti-asialoGM1 Ab treatment of mice, which specifically depletes NK cells, greatly increased the number of metastases of colon cancers, NK cells were considered to be the main antimetastatic effectors [46]. Interestingly, when a OK432 was injected i.v. into mice, NK cells increased in the liver, and the antimetastatic function of the liver MNCs against colon cancers greatly increased [46]. This was also the case for *α*-GalCer and liver NK cells. However, as described above, the administration of either IL-12 or LPS activates NKT cells and inhibits tumor metastasis in the liver. Therefore, NK cells and NKT cells either independently or cooperatively act as antitumor effectors both in mice and humans. However, the antitumor effects of Kupffer cells themselves are controversial. Although the cytokines produced by Kupffer cells (IL-12, IFN-*α*) are indeed important for the activation of NK cells and NKT cells and for preventing tumor liver metastases, depletion of Kupffer cells by gadolinium chloride or clodronate liposomes increased the number of liver metastasis in some reports [82, 83] while it did not affect the number of tumor metastases in the liver in other reports [46]. In vitro experiments also showed that Kupffer cells can phagocytose tumor cells and can kill them [84], although another report contradicted this claim [85].

Our unpublished observations showed that NK cells and NKT cells in the human liver tissues close to metastatic colon tumors express less perforin than those in the liver tissues distant from metastatic tumors, implying that tumor metastasis starts to grow at the area where lymphocyte activity is attenuated. Alternatively, tumors may produce paracrine factors which may inhibit perforin production and antitumor cytotoxicity of NK/NKT cells around tumors.

## 11. Concluding Remarks

The liver contains innate immune effectors, Kupffer cells, NK cells, NKT cells, and CD8^+^CD122^+^ cells, and these cells cooperatively act not only against bacterial and viral infections but also against cancers. Many bacterial components and toxins from the portal vein and systemic circulation activate Kupffer cells to produce IL-12 and induce potent antitumor activity by NK cells, NKT cells, and CD8^+^CD122^+^ cells via IFN-*γ*/perforin/granzyme pathway (Figure 2). CD16^−^CD56^++^ NK cells in PBMC and presumably in the liver of humans may also play an important role in antitumor immunity, infections, and some autoimmune diseases. The IFN-*γ* produced by these innate immune lymphocytes in the liver in turn stimulates the phagocytic activity and cytokine production of Kupffer cells via a positive feedback loop (Figure 2). Liver NK cells, NKT cells, and CD8^+^CD122^+^ cells may also migrate to other organs to inhibit tumor growth there. Decreased NKT cells and NK cells in cirrhotic livers in hepatitis C patients may therefore allow for the development of HCC. However, the TNF produced by Kupffer cells and TNF-activated liver lymphocytes, NKT cells and NK cells, may be responsible for septic shock, hepatocyte injury/regeneration, cholangiocyte injury, and MODS via the TNF/FasL/Fas pathway (Figure 2).

## Figures and Tables

**Table 1:** NKT cells are IL-12-induced antimetastatic effectors.

Mouse strain/treatment	Tumor	Site of metastasis tested	Number of tumor metastases		
			Control	IL-12 treated	% inhibition
BALB/c + IL-12	RL♂1	liver	216 ± 24	28 ± 2	87%*
	Colon 26	lung	125 ± 25	16 ± 10	87%*
DBA/2 + lL-12	P815	liver	173 ± 12	10 ± 1	94%*
C57BL/6 + IL-12	B16	lung	61 ± 16	5 ± 1	91%*
	EL4	liver	106 ± 22	17 ± 7	84%*
C57BL/6 bg/bg + IL-12	EL4	liver	107 ± 17	16 ± 6	85%*

C57BL/6 + IL-12	EL4	liver	96 ± 18	15 ± 4	84%*
	3LL	lung	122 ± 26	25 ± 5	80%*
C57BL/6 + *α*AGM1 Ab + IL-12	EL4	liver	102 ± 24	22 ± 5	78%*
	3LL	lung	128 ± 32	33 ± 8	74%*
C57BL/6 + *α*NK1.1 Ab + IL-12	EL4	liver	152 ± 26	130 ± 20	14%
	3LL	lung	204 ± 36	156 ± 28	24%

The mice were inoculated i.v. with syngeneic tumors. Data of tumor metastasis and % of inhibition are shown as mean ± SD from six to ten mice in each group. *P < .01. *α*AGM1 Ab: antiasialo GM1 antibody; *α*NK1.1 Ab: anti-NK1.1 antibody.

**Table 2:** Antitumor or hepatotoxic effectors in the liver.

Function	Reagents				
	IL-12	*α*-GalCer	LPS	OK432	CpG
Antitumor effectors	NKT	NK	NKT	NK	NK
IFN-*γ* producers	NKT	NKT(NK)	NK	NK	NK
Hepatotoxic effectors	NKT	NKT	NKT/NK	?	NKT
